# Prevalence of asymptomatic left ventricular systolic dysfunction in hypertensive Nigerians: echocardiographic study of 832 subjects

**DOI:** 10.5830/CVJA-2010-063

**Published:** 2011-11

**Authors:** OS Ogah, RO Akinyemi, JO Adesina, JKL Osinfade, RF Ogundipe, GD Adegbite, OI Udofia, SB Udoh, OS Ojo, AA Alabi, T Majekodunmi, AO Falase

**Affiliations:** Department of Medicine, Federal Medical Centre, Idi-Aba, Abeokuta, Ogun State, Nigeria; Department of Medicine, Federal Medical Centre, Idi-Aba, Abeokuta, Ogun State, Nigeria; Department of Medicine, Federal Medical Centre, Idi-Aba, Abeokuta, Ogun State, Nigeria; Department of Medicine, Federal Medical Centre, Idi-Aba, Abeokuta, Ogun State, Nigeria; Department of Medicine, Federal Medical Centre, Idi-Aba, Abeokuta, Ogun State, Nigeria; Department of Medicine, Sacred Heart Hospital Lantoro, Abeokuta, Ogun State, Nigeria; Department of Medicine, Sacred Heart Hospital Lantoro, Abeokuta, Ogun State, Nigeria; Department of Medicine, Sacred Heart Hospital Lantoro, Abeokuta, Ogun State, Nigeria; Department of Medicine, Sacred Heart Hospital Lantoro, Abeokuta, Ogun State, Nigeria; Department of Medicine, Sacred Heart Hospital Lantoro, Abeokuta, Ogun State, Nigeria; Division of Adult Congenital Heart Disease, Department of Cardiology, Royal Brompton Hospital, London, United Kingdom; Department of Medicine, University of Ibadan, Oyo State, Nigeria

**Keywords:** hypertension, echocardiography, systolic dysfunction, Nigeria

## Abstract

**Background:**

We sought to determine the prevalence of echocardiographically determined left ventricular systolic dysfunction in asymptomatic hypertensive subjects seen in Abeokuta, Nigeria.

**Methods:**

Echocardiography was performed in 832 consecutive hypertensive subjects referred for cardiac evaluation over a three-year period.

**Results:**

Data were obtained in 832 subjects (50.1% women) aged 56.0 ± 12.7 years (men 56.9 ± 13.3 years, women 55.0 ± 12.0 years, range 15–88). The prevalence of left ventricular systolic dysfunction (LVSD) was 18.1% in the study population (mild LVSD = 9.6%, moderate LVSD = 3.7% and severe LVSD = 4.8%). In a multivariate analysis, male gender, body mass index and LV mass were the predictors of LVSD.

**Conclusion:**

Significant numbers of hypertensive subjects in this study had varying degrees of left ventricular systolic dysfunction. Early introduction of disease-modifying drugs in these patients, such as angiotensin converting enzyme inhibitors or angiotensin receptor blockers may retard or prevent the progression to overt heart failure.

## Abstract

High blood pressure affects about a billion people worldwide.^1^-^3^ It has been predicted that by 2025, more than 1.5 billion adults will have hypertension.^1^-^3^ In the year 2001, hypertension was estimated to be responsible for 7.6 million premature deaths worldwide (13.5% of total global mortality) and it was also responsible for 92 million disability-adjusted life years (DALYs).^1^-^3^ The condition has been rightly described as the foundation of cardiovascular disease in sub-Saharan Africa.^4^ The overall prevalence has been put at 10 to 15% but rates as high as 30 to 32% have been reported.^5^

Hypertension and its complications are responsible for about 25% of urban hospital medical admissions in Nigeria,^6^ as well as for over 80% of cardiac clinic consultations in the country. It is the most frequently diagnosed medical illness in elderly populations^7^ and senior executives.^8^

High blood pressure is also by far the commonest cause of chronic renal failure, stroke, heart failure and sudden unexpected death in Nigeria.^9^

In Nigeria, the prevalence of asymptomatic left ventricular systolic dysfunction (LVSD) among people with hypertension is unknown. The aim of the study was therefore to determine the prevalence of asymptomatic LVSD in hypertensive subjects in Abeokuta, Nigeria.

## Methods

The study was conducted at the Federal Medical Centre (FMC), Idi-Aba and the Sacred Heart Hospital (SHH), Lantoro, both in Abeokuta, the capital city of Ogun State in south-western Nigeria. FMC was established in 1993 by the federal government of Nigeria to cater for the health needs of the people of Ogun State and its environs in south-western Nigeria. The state has a population of about 3.2 million and a surface area of about 16 409.26 km^2^. SHH is one of the oldest hospitals in Nigeria, established in 1897 by the German Catholic Mission.

Cardiological services commenced in the city in September 2005 and since then a registry of patients and services rendered has been kept. A cardiologist (OSO) covers the two cardiac units, which are about 5 km apart, assisted by senior medical officers and postgraduate resident doctors and well as nurses. Facilities available for cardiac evaluation include chest radiography, 12-lead electrocardiography (ECG), exercise ECG, Holter ECG, ambulatory blood pressure-monitoring devices, spirometry and echocardiography (ECHO).

This was a cross-sectional study, conducted within a three-year period. Hypertensive patients were eligible for the study if they fulfilled the following criteria: (1) no evidence of valvular abnormality. This was based on clinical examination and absence of features of valvular heart disease at echocardiography; (2) absence of congestive heart failure based on previous history of admission for heart failure or symptoms and signs of heart failure in the past or at the time of evaluation, using the Framingham criteria; (3) absence of sickle cell disease based on self-reported haemoglobin electrophoretic pattern of the subject and/or absence of stigmata of the disease; (4) absence of self-reported history of renal failure or serum creatinine ≥ 2 mg/dl; (5) absence of ischaemic heart disease based on history, as well as absence of ischaemic ECG changes at the time of the study (other than left ventricular hypertrophy with strain pattern); (6) other exclusion criteria included morbid obesity, pulmonary heart disease (cor pulmonale), chest abnormality that obscured echo-window and left bundle branch block pattern on the 12-lead ECG.

**Fig. 1:**
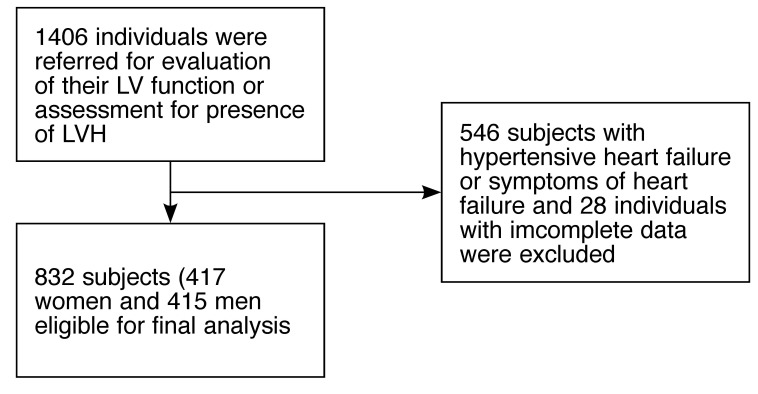
Criteria for selection of the subjects for analysis.

Using a simple questionnaire, a nurse screened the patients for history or symptoms of these and history of previous hospital admissions relating to the exclusion criteria. All the echocardiography request forms were also assessed for evidence of exclusion criteria. Fig. 1 shows how the patients were selected for final analysis. Both treated and untreated hypertensive subjects were recruited. Hypertension was defined according to international criteria.^10^

Baseline clinical and demographic characteristics were obtained from the subjects. These included date of birth, age, gender and history of diabetes. Blood pressure measurements were obtained according to standard guidelines,^11^ with a mercury sphygmomanometer (Accosson London). Systolic and diastolic blood pressures were measured at Korotkoff sounds phases I and V, respectively. Blood pressure was measured three times on the right arm, after a five-minute rest, and averaged. Subjects were weighed without shoes and in light clothing on a standard beam balance. Height was measured to the nearest centimetre using an anthropometrical plane with the subjects without shoes or headgear. Body mass index (BMI) was calculated using the formula: BMI = weight (kg)/height (m)^2^. Body surface area (BSA) was calculated using the formula of Dubois.^12^

M-mode, two-dimensional (2-D) and Doppler echocardiography were performed using a standard protocol and an ALOKA SSD 4000 echocardiography machine (Aloka Co, Ltd, Tokyo, Japan). Two-dimensional guided M-mode measurements were made according to the recommendations of the American Society of Echocardiography (ASE).^13^ Left ventricular internal dimension, posterior wall thickness and interventricular septal thickness were measured at end-diastole and end-systole. Where optimal M-mode imaging could not be obtained, 2-D linear measurements were obtained according to the ASE criteria.^13^ Left atrial end-systolic diameter was obtained from the trailing edge of the posterior aortic–anterior left atrial complex. Measurements were obtained in up to three cardiac cycles, according to the ASE convention.^13^

One experienced cardiologist performed all the echocardiography. In our laboratory, the intra-observer concordance correlation coefficient and measurement error have been reported.^14^

Left ventricular systolic performance (LVSP) was assessed using the fractional shortening of the left ventricle and the ejection fraction. Left ventricular ejection fraction (LVEF) was calculated using the Teichholz formula.^15^ Fractional shortening was calculated from LV internal dimensions in diastole and systole:

Fractional shortening = $\frac{LVIDd\;–\;LVIDs}{LVID}\times 100$

Left ventricular mass (LVM) was calculated using the formula of Devereux and Reichek.^16^ This has been shown to yield LVM closely related to autopsy measurements (*r* = 0.90)^17^ and it had good inter-observer reproducibility (*p* = 0.93) in one study.^18^ Relative wall thickness (RWT) was derived from 2 × posterior wall thickness/LV internal diameter.

Left ventricular hypertrophy (LVH) was defined by LV mass indexed by allometric signal (height^2.7^) > 51 g/m^2.7^.^19^ This partition value of 51 g/m^2.7^ was used since this was the only criterion that demonstrated as the optimal threshold value for left ventricular hypertrophy in blacks, irrespective of gender, in two previous studies. Left ventricular systolic function was categorised as follows:^20^ normal LV function, EF ≥ 50%; mild LVSD, EF 40–49%; moderate LVSD, EF 30–39%; and severe LVSD, EF < 30%.

## Statistical analysis

SPSS version 11.0 software (SPSS, Chicago, IL, USA) was used in the analysis of the data. Continuous variables were expressed as mean ± SD, while categorical variables were expressed as counts (percentages). Comparison between the two groups was assessed with the Student’s *t*-test for independent variables, while the χ^2^ analysis was used to compare proportions. Analysis of variance (ANOVA) with Scheffe’s *post hoc* test was used for the comparison of multiple groups. A two-tailed *p*-value of 0.05 was assumed statistically significant.

## Results

A total of 832 subjects, 415 men (49.9%) and 417 women (50.1%) were eligible for analysis. Fig. 1 shows the criteria for selection of subjects for analysis.

**Fig. 2:**
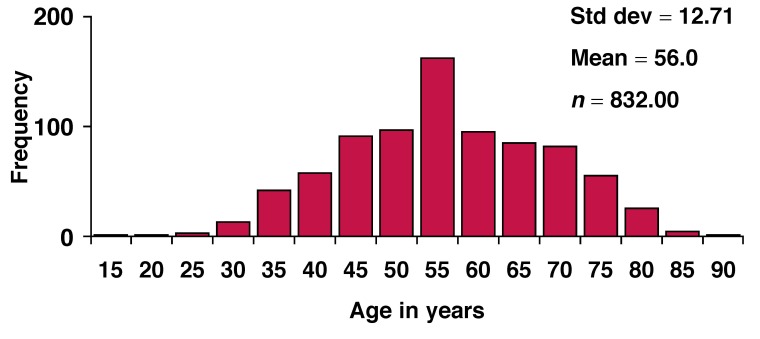
Histogram of the age distribution of the subjects.

The overall mean age of the population was 56.0 ± 12.7 years (range 15–88). Fig. 2 is a histogram showing the age distribution of the subjects. The majority of the subjects fell within the middle-aged group, with a peak age of 50 years. Forty-six subjects (5.5%) had a self-reported history of diabetes (25 men, 6.0% and 21 women, 5.0%).

Tables 1 and 2 show the clinical and demographic characteristics of the subjects in the men and women, respectively. Patients with LVSD were older. Those with normal left ventricular systolic function (LVSF) were heavier and had a larger body surface area. Diastolic blood pressure as well as mean arterial blood pressure were higher in individuals with severe LVSD.

**Table 1:** Clinical And Demographic Characteristics Of The Male Subjects

*Parameter*	*Normal LVSF*	*Mild LVSD*	*Moderate LVSD*	*Severe LVSD*	*ANOVA p-value*
Age (years)	56.4 (13.8)	62.7 (9.0)	58.9 (12.5)	61.5 (16.8)	0.039
Weight (kg)	77.9 (15.5)	72.3 (14.8)*	63.0 (14.8)*	68.2 (20.0)*	0.0001
Height (cm)	169.0 (7.4)	169.8 (5.3)	166.4 (9.1)	167.5 (8.2)	NS
BMI (kg/m^2^)	27.3 (5.0)	25.0 (4.7)*	22.6 (5.9)*	24.3 (7.0)*^∞^	0.0002
BSA (m^2^)	1.88 (0.19)	1.83 (0.18)*	1.69 (0.24)*^∞^	1.76 (0.23)*	0.0001
Systolic BP (mmHg)	84.5 (14.9)	81.2 (11.1)	83.4 (12.4)	88.9 (13.8)	NS
Diastolic BP (mmHg)	91.0 (14.9)	84.2 (12.7)*	85.6 (11.5)	99.0 (15.9)*^∞^	0.018
Pulse pressure (mmHg)	57.0 (17.3)	57.8 (17.3)	59.4 (19.5)	53.9 (16.1)	NS
MAP (mmHg)	110.0 (15.9)	103.4 (14.7)*	105.4 (11.9)	116.9 (29.0)^∞^	0.024

BMI = body mass index, BSA = body surface area, BP = blood pressure, MAP = mean arterial pressure. NS = not significant, *p < 0.05 versus normal LVSF, ^∞^*p* < 0.05 versus mild LVSD.

**Table 2:** Clinical And Demographic Characteristics Of The Female Subjects

*Parameter*	*Normal LVSF*	*Mild LVSD*	*Moderate LVSD*	*Severe LVSD*	*ANOVA p-value*
Age (years)	56.4 (13.3)	60.1 (10.1)	60.1 (13.5)	55.4 (17.0)	NS
Weight (kg)	76.2 (14.9)	74.5 (154.6)	64.2 (16.4)	71.0 (19.9)	NS
Height (cm)	168.4 (7.5)	169.2 (9.4)	166.3 (9.1)	167.6 (7.7)	NS
BMI (kg/m^2^)	26.9 (4.9)	26.4 (7.8)*	23.1 (5.4)^∞^	25.2 (6.3)^∞^	0.007
BSA (m^2^)	1.86 (0.19)	1.84 (0.17)	1.71 (0.22)	1.79 (0.24)*^∞^	0.001
Systolic BP (mmHg)	147.2 (22.3)	142.0 (21.7)	143.2 (19.1)	148.5 (33.5)	NS
Diastolic BP (mmHg)	90.4 (14.7)	85.1 (12.5)*	85.5 (11.0)	98.5 (24.9)	0.002
Pulse pressure (mmHg)	57.4 (16.5)	57.6 (17.1)	58.5 (18.7)	52.1 (17.5)	NS
MAP (mmHg)	110.2 (16.0)	103.5 (14.5)	105.5 (11.3)	116.3 (28.2)	0.023

BMI = body mass index, BSA = body surface area, BP = blood pressure, MAP = mean arterial pressure. NS = not significant, *p < 0.05 versus normal LVSF, ^∞^*p* < 0.05 versus mild LVSD.

**Fig. 3:**
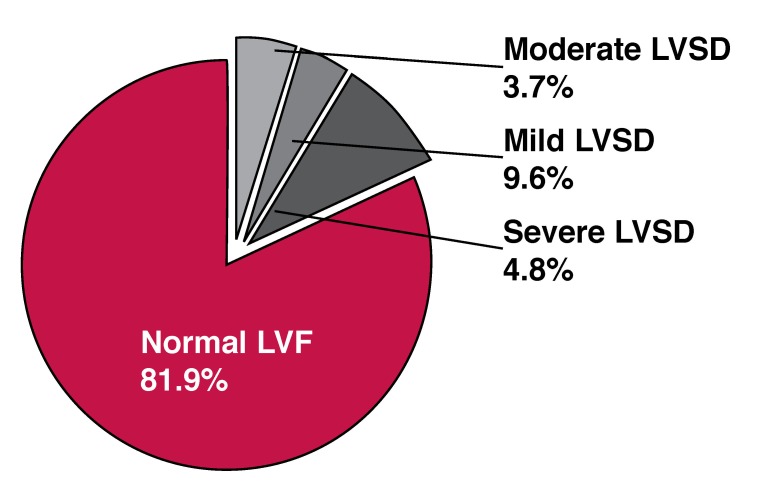
Distribution of LVS F.

**Fig. 4:**
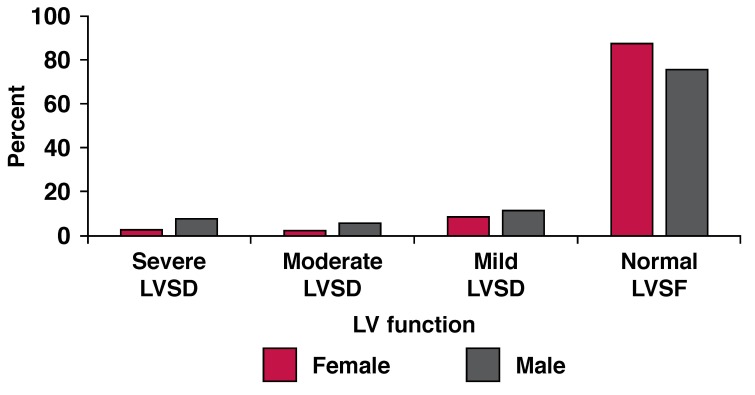
Distribution of LVS F according to gender.

Normal LVSF, defined as ejection fraction (EF) ≥ 50% was present in 89.9% of the subjects. The remaining 18.1% had LVSD (mild 9.6%, moderate 3.7% and severe 4.8%). Figs 3 and 4 depict the distribution of the subjects according to LVSF and gender.

Men had a higher prevalence of LVSD than women (24.4 and 12.3%, respectively) and this was statistically significant (*p* < 0.0001). Compared with individuals with normal LVSF or mild LVSD, those with severe LVSD were older, more likely to be men, had larger left atrial diameter, larger LV dimensions and LV mass, and as expected, lower fractional shortening and ejection fraction. This finding was consistent in both genders and progressed from normal LVSF to severe LVSD (Tables 3, 4).

**Table 3:** LV Findings In Male Subjects According To Functional Status

*Parameter*	*Normal LVSF*	*Mild LVSD*	*Moderate LVSD*	*Severe LVSD*	*ANOVA p-value*
AO diameter (cm)	3.10 (0.44)	3.25 (0.41)*	3.07 (0.39)	3.15 (0.45)	NS
LA diameter (cm)	3.78 (0.58)	4.03 (0.58)*	4.34 (0.79)*	4.45 (0.67)*	< 0.0001
IVSd (cm)	1.20 (0.31)^∞^	1.32 (0.25)	1.13 (0.23)^∞^	1.20 (0.34)	NS
IVSs (cm)	1.59 (0.39)	1.47 (0.33)	1.30 (0.16)*	1.41 (0.48)*	0.0003
LVIDd (cm)	4.67 (0.69)	5.24 (0.86)*	6.11 (0.98)*^∞^	6.06 (1.11)*^∞^	< 0.0001
LVIDs (cm)	3.01 (0.61)	4.15 (0.88)*	5.07 (0.87)*^∞^	5.20 (1.13)*^∞^	< 0.0001
PWTd (cm)	1.28 (0.36)	1.27 (0.41)	1.29 (0.31)	1.32 (0.41)	NS
PWTs (cm)	1.85 (0.38)	1.66 (0.37)*	1.61 (0.39)*^∞^	1.66 (0.43)^∞^	0.0003
FS (%)	35.7 (8.0)	21.1 (7.1)*	17.0 (4.5)*^∞^	14.4 (8.3)*^∞^	< 0.0001
EF (%)	65.9 (9.3)	45.0 (2.8)*	33.6 (2.5)*^∞^	23.5 (7.0)*^∞^	< 0.0001
LVM (g)	221.0 (91.2)	286 (121.6)*	337.1 (122.1)*^∞^	356.4 (169.6)*^∞^	< 0.0001
LVM/BSA (g/m^2^)	119.6 (48.6)	155.3 (62.9)*	201.8 (78.3)*^∞^	203.0 (99.9)*^∞^	< 0.0001
LVM/HT^2.7^ (g/m^2.7^)	54.3 (22.1)	68.9 (27.0)*	85.6 (29.8)*^∞^	89.7 (45.1)*^∞^	< 0.0001

AO = aortic root, LA = left atrium, IVSd = interventricular septum in diastole, IVSs = interventricular septum in systole, LVIDd = left ventricular internal diameter in diastole, LVIDs = left ventricular internal diameter in systole, PWTd = posterior wall thickness in diastole, PWTs = posterior wall thickness in systole, FS = fractional shortening, EF = ejection fraction, LVM = left ventricular mass, BSA = body surface area, HT = height. NS = not significant, *p < 0.05 vs normal LVSF, ^∞^*p* < 0.05 vs mild LVSD.

**Table 4:** LV Findings In Female Subjects According To Functional Status

*Parameter*	*Normal LVSF*	*Mild LVSD*	*Moderate LVSD*	*Severe LVSD*	*ANOVA p-value*
AO diameter (cm)	3.08 (0.44)	3.22 (0.42)	3.07 (0.39)	3.15 (0.45)	NS
LA diameter (cm)	3.75 (0.58)	4.01 (0.56)*	4.34 (0.79)*	4.45 (0.67)*	< 0.0001
IVSd (cm)	1.20 (0.31)^∞^	1.33 (0.25)	1.13 (0.23)^∞^	1.20 (0.34)	0.027
IVSs (cm)	1.57 (0.38)	1.47 (0.32)	1.30 (0.16)*	1.20 (0.34)*	0.0006
LVIDd (cm)	4.63 (0.71)	5.25 (0.87)*	6.11 (1.00)*	6.06 (1.11)*	< 0.0001
LVIDs (cm)	2.99 (0.62)	4.17 (0.85)*	5.07 (0.87)*	5.20 (1.13)*	< 0.0001
PWTd (cm)	1.27 (0.52)	1.24 (0.40)	1.29 (0.31)	1.66 (0.43)	NS
PWTs (cm)	1.84 (0.38)	1.67 (0.36)*	1.61 (0.39)*	1.66 (0.43)	0.0003
FS (%)	35.6 (7.9)	21.0 (6.6)*	17.0 (4.5)*	14.4 (8.3)*	< 0.0001
EF (%)	65.9 (9.3)	44.7 (2.7)*	33.5 (2.5)*	23.5 (2.5)*^∞^	< 0.0001
LVM (g)	145.7 (38.3)*	174.3 (73.0)*	198.1 (77.5)	218.3 (48.8)*	< 0.0001
LVM/BSA (g/m^2^)	113.5 (45.9)	128.3 (48.8)	145.7 (38.3)*	174.3 (73.0)*^∞^	0.0001
LVM/HT^2.7^ (g/m^2.7^)	56.5 (22.5)	64.023.6)*	73.4 (18.2)*	81.8 (30.5)*^∞^	< 0.0001

AO = aortic root, LA = left atrium, IVSd = interventricular septum in diastole, IVSs = interventricular septum in systole, LVIDd = left ventricular internal diameter in diastole, LVIDs = left ventricular internal diameter in systole, PWTd = posterior wall thickness in diastole, PWTs = posterior wall thickness in systole, FS = fractional shortening, EF = ejection fraction, LVM = left ventricular mass, BSA = body surface area, HT = height. NS = not significant, *p < 0.05 vs normal LVSF, ^∞^*p* < 0.05 vs mild LVSD.

We developed a linear regression model for the whole population (as the pattern was similar in both genders). LVEF was used as the dependent variable, while independent variables were taken from clinical variables (age, gender, body mass index, body surface area) and echocardiographic variables [left atrial diameter, LV internal dimensions, LV mass, relative wall thickness (RWT)] that were significant in the univariate analysis.

Although LV dimensions were significantly related to LVEF in the univariate analysis, they were not added in the final model since these parameters were used in the determination of LVM. Categorical variables such as gender and presence or absence of diabetes mellitus were entered as indicator variables. None of the blood pressure variables were significant in the univariate analysis.

In the univariate analysis, EF was related to age [B (unstandardised regression coefficients) = –0.84, β (standardised regression coefficient) = –0.72, *p* = 0.036]; gender (B = –5.42, β = –1.84, *p* < 0.0001); BMI (B = 0.31, β = 0.13, *p* = 0.0002); left atrial diameter (B = –5.72, β = –0.25, *p* < 0.0001); end-diastolic diameter (B = –7.70, β = –0.45, *p* < 0.0001), end-systolic diameter (B = –11.97, β = –0.76, *p* ≤ 0.0001); LVM (B = –0.48, β = –0.32, *p* < 0.0001); and RWT (B = 14.38, β = 0.19, *p* < 0.0001).

In a multivariate analysis, lower LVEF was independently related to BMI, gender, LV mass, left atrial diameter and relative wall thickness.

## Discussion

Hypertension is the commonest cardiovascular disease in Nigeria and sub-Saharan Africa. It is the commonest risk factor for heart failure, stroke and chronic renal impairment. In Nigeria and most African countries, the majority of patients first present with evidence of target-organ damage such as overt heart failure. Most often, overt heart failure is preceded by asymptomatic left ventricular systolic dysfunction. Information on the prevalence and burden of both symptomatic and asymptomatic LVSD is based on population studies as well as studies done in hypertensive subjects in Europe and America.^21^-^23^

The burden of LVSD (symptomatic or asymptomatic) in hypertensive subjects is largely unknown in Nigeria and in most African countries, hence the reason for the present study. Echocardiographic methods have been shown to provide a high yield of quantitative measurement of LV function and can be used to assess the determinants of LV function, both in hypertensive and general populations.

The main findings of the study are: the majority of our hypertensive patients fell within the middle-aged group with a peak age of 50 years, about 18% of our hypertensive subjects had asymptomatic LVSD, the prevalence of LVSD was higher in men than in women, LVSD increased with age, although LVSD was independent of age, and the main predictors of LVSD were male gender, body mass index, left ventricular mass and LV relative wall thickness.

All over sub-Saharan Africa, the peak age of presentation of cardiovascular diseases such as hypertension has been well defined.^24^-^26^ As in the present study, most patients are within the age group of 40 to 60 years, often with a peak age of 45 to 50 years, as observed in this study. The implication is that where complications arise, it is usually associated with high DALYs, with a huge impact on the socio-economic growth of the family and nation, as the population affected is in the prime of life. This is contrary to the situation in developed countries where most cardiovascular diseases manifest after the age of 65 years.

In a univariate analysis, age was related to LVEF. However, when other factors such as gender, LV mass, relative wall thickness and BMI were factored in, the association with age became insignificant. The plausible reason for this is that the impact of age on LVSF is probably mediated through other factors. Our finding is similar to the report of other workers.^22^,^23^,^27^,^28^

The study shows that the prevalence of impaired LVSF is about twice as common in men as in women. In a multivariate analysis, male gender was found to be an independent predictor of impaired LVSF. This is similar to the findings of authors in studies done in hypertensive subjects or in the general population.^21^-^23^,^28^ The reasons for this are not clear but it is well known that cardiovascular diseases generally occur earlier in men than in women. In our setting, women are also more likely to attend follow-up clinics as well as take their medication as prescribed.

Tables 1 and 2 show that diastolic blood pressure and mean arterial blood pressure were higher in the group with severe LVSD compared to other groups. No significant difference was found in the systolic blood pressure and pulse pressure among the groups.

In univariate and multivariate analyses, blood pressure was not found to be related to LVSF. This finding is similar to that of Devereux *et al*.,^22^ but at variance with studies done in the general population, where the relationship of blood pressure with LVSF persisted in the multivariate analysis.^21^,^29^,^30^ This may be may be due to the fact that most of our patients were on antihypertensive medications.

In this study, BMI was found to decrease from subjects with normal LVSF to those with severe LVSD (although the lowest BMI was in those with moderate LVSD in both men and women). The impact of BMI persisted in the multivariate analysis. This relationship between LVSF and BMI was also reported by Devereux *et al*.^22^ Because our population was relatively lean, one plausible reason for the finding may be the known relationship between chronic LVSD and weight loss (cardiac cachexia).

In a univariate analysis, we did not find any relationship between LVSD and the presence of diabetes in this study. This is at variance with the report by some workers.^21^,^22^ The reason for the negative finding in our study may be that we relied on self-reported history of diabetes. Many more subjects with diabetes could have been detected if metabolic profiles were run in our study subjects.

The left atrial size was found to be independently related to LVSF. The larger the left atrium, the poorer the LVSF. Similar findings have been reported by other investigators.^31^,^32^ Left atrial size is a known strong marker or surrogate of left diastolic function. The latter is known to have a positive relationship with LVSF.^33^,^34^

## Limitations of the study

We noted the following limitations with this study. It was a hospital-based study and may not reflect the situation in the general population. We did not run metabolic profile functions (blood glucose, lipid, uric acid, insulin levels, etc) in the present study, as was done in studies in industrialised nations, due to lack of funds.

Newer measures of assessment of LVSF such as LV mid-wall shortening, circumferential end-systolic measurements as well as measures of arterial wall stiffening (pulse pressure/stroke index) were not assessed in our study. Absence of ischaemic heart disease was only assessed based on clinical history and 12-lead ECG. This may not exclude sub-clinical ischaemic heart disease.

## Conclusions

LVSD, assessed as ejection fraction < 50%, was detected in 18.1% of our hypertensive population, who did not have symptoms of overt heart failure. Male gender, body mass index, LVM and relative wall thickness were found as independent predictors of LVSF in our study.

Significant numbers of hypertensive subjects in this study had varying degrees of left ventricular systolic dysfunction. Early introduction of disease-modifying drugs in these patients, such as angiotensin converting enzyme inhibitors/angiotensin receptor blockers may retard or prevent the progression to overt heart failure.

It must be stated that since echocardiography is expensive, it cannot be placed as the first line of systematic screening of hypertensive patients, however, the predictive factors of LVSD are accessible to primary-care physicians. For instance, the combination of male gender and obesity should draw the attention of clinicians to the possible presence of LVSD, a pathogenic precursor of heart failure.
